# Prevalence and impact of misdiagnosed drug allergy labels among patients with hereditary angioedema

**DOI:** 10.3389/falgy.2022.953117

**Published:** 2022-08-16

**Authors:** Jane Chi Yan Wong, Noel Cheong, Chak Sing Lau, Philip Hei Li

**Affiliations:** Division of Rheumatology & Clinical Immunology, Department of Medicine, The University of Hong Kong, Hong Kong, Hong Kong

**Keywords:** hereditary angioedema, prevalence, drug allergy, Hong Kong, drug delabelling

## Abstract

**Introduction:**

Hereditary angioedema (HAE) is a rare condition with presents with episodic attacks of angioedema, which is often misdiagnosed as allergy, and associated with significant morbidity and mortality. Misdiagnosed drug allergy (DA) labels are also associated with a multitude of adverse clinical outcomes. However, the prevalence and impact of incorrect DA labels on HAE remains unknown.

**Methods:**

Data from the clinical records of all HAE patients in Hong Kong were collected and analysed. All HAE patients with DA labels on their medical records were recruited to proceed with DA testing, including confirmatory drug provocation tests (DPT).

**Results:**

Nine (22%) out of a total of 41 HAE patients carried at least one DA label. Five of nine (56%) patients had more than 1 DA label and there was a total number of 16 DA labels. The most common DA label was to beta-lactams (37.5%). Presence of DA label was associated with delay in HAE diagnosis (23.8 ± 11.1 vs. 10.2 ± 14.3 years, *p* = 0.012), likelihood of HAE attacks (100% vs. 46.9%, *p* = 0.005) and rate of hospitalization (3.78 ± 2.68 vs. 1.32 ± 2.61, *p* = 0.022) per year. All (100%) of all DA labels were disproven and removed after confirmatory DPT were performed.

**Conclusion:**

DA labels are prevalent among HAE patients but are frequently misdiagnosed and mislabelled. Misdiagnosed DA are associated with delay in HAE diagnosis as well as adverse clinical outcomes. Immunologists/allergists should consider pre-emptively reviewing and investigate every suspicious DA label, especially among HAE patients.

## Introduction

Hereditary angioedema (HAE) is rare primary immunodeficiency caused by a C1 inhibitor protein (CI-INH) deficiency or dysfunction resulting in recurrent bradykinin-mediated angioedema (1). As an uncommon cause of angioedema, HAE is frequently misdiagnosed as chronic spontaneous urticaria or various “allergies”, leading to significant delay in diagnosis (2, 3). Drug allergies (DA) are also frequently mislabelled during HAE attacks, especially when attacks have been triggered by intercurrent illnesses and patients were prescribed medications shortly prior.

Every 1 in 15 of the population of Hong Kong have at least one physician reported DA label; most commonly to beta-lactams (BL) antibiotics, non-BL antibiotics and non-steroidal anti-inflammatory drugs (NSAID) (4–7). Many of these DA labels, especially for BL, are found to be inaccurate after formal allergy workup and have been associated with a variety of adverse clinical outcomes (4–6, 8–10). Due to the lack of Specialists in Immunology & Allergy in Hong Kong, suspected DA are seldom investigated or revisited and most patients live with misdiagnosed “allergy” labels for the rest of their lives (11, 12). This problem is likely compounded in HAE patients, who have accrued multiple misdiagnosed DA labels (especially prior to their diagnosis of HAE) and are prone to recurrent hospital admissions (2).

However, to our knowledge, there have been no prior studies investigating the burden and impact of DA labels in HAE patients. The potential benefit of delabelling false DA labels among HAE patients is also unknown. We therefore investigated the prevalence and impact of delabelling false DA labels by performing complete allergological tests workup for all HAE patients with DA labels in Hong Kong.

## Methods

### Patient recruitment

Patients were identified by reviewing electronic medical records of all patients diagnosed with HAE in Queen Mary Hospital (QMH), Hong Kong between 2016 and 2021 with labelled drug allergies. Queen Mary Hospital is the only referral center with specialist Immunology & Allergy services under the Hong Kong's public health system and receives referrals from across the whole territory. Healthcare services in Hong Kong are mainly provided by the public sector and QMH has close liaison with private immunologists across the territory as well as the local HAE patient support group (hae hk). QMH is also the only local immunology laboratory in Hong Kong to offer C1-inhibitor level and function testing, receiving all requests for diagnostic confirmation. All confirmed cases of HAE have been referred to QMH for subsequent management. Therefore, this cohort likely represents all the patients diagnosed with HAE in Hong Kong.

### Drug allergy workup

All HAE patients with DA labels on their medical records were recruited to proceed with DA testing. Only drugs which the index formulation was known and available in the hospital formulary, or when re-exposure to the drugs was not contraindicated were included. All patients with a BL allergy were referred for further allergy workup including skin tests (ST) and drug provocation test (DPT) performed in accordance with the British Society for Allergy and Clinical Immunology and Hong Kong Institute of Allergy guidelines (13). Other DA workup was performed in accordance to the International Consensus on Drug Allergy (14). Informed written consent was obtained for all patients. All DPT were carried out under strict hospital surveillance. Only those patients who completed a negative DPT were considered non-allergic to their respective drug(s).

### Data collection

Baseline characteristics including age, sex, type of HAE (I or II), presence, frequency and severity of symptoms, age of diagnosis, delay in diagnosis (number of years between first HAE attack and diagnosis), number of admissions per year, number of HAE attacks per year and whether the patient has ever been hospitalized due to HAE were obtained. “Frequent” HAE attacks were defined as ≥5 attacks per year. All DA labels were physician-reported and retrieved through the electronic record system. Details including the index drug(s), suspected type of hypersensitivity reaction (immediate [symptom onset within 1 h of drug exposure], non-immediate [symptom onset after 1 h of drug exposure] or unknown], whether the DA labels were given before or after diagnosis of HAE, and number of years of carrying a DA label were obtained. For patients with BL allergy, use of alternative antibiotics (class, route of administration, days of administration), days of hospitalization due to infections and if available, microorganisms during infective episodes and their antibiotic sensitivity patterns were taken.

### Statistical analysis

Categorical variables are expressed as number (percentages) and continuous variables were reported as mean (2 standard deviations) and median (range) when appropriate. Fisher's exact test statistic and independent samples *t*-test were used to compare categorical and continuous variables between groups respectively. A *p* value of less than 0.05 was considered statistically significant. SPSS Statistics version 26 (IBM Corp., Armonk, NY, USA) was used for all the analyses and figures.

### Ethics

All patients gave informed consent to participate in this study. This study was approved by the Institutional Review Board of the University of Hong Kong/Hospital Authority Hong Kong West Cluster. 

## Results

### Demographics and clinical characteristics

A total of 41 patients were diagnosed with HAE in Hong Kong, and their demographics and clinical characteristics are shown in Table 1. Three DA labels, including: “traditional Chinese medicine” (unable to retrieve formulation), “oral contraceptive pill” (relatively contraindicated for HAE patients) and “contrast” (unable to identify index contrast used) were excluded for analysis. Nine (22%) HAE patients carried at least one DA label, this prevalence is higher than that of the general population (14%) and comparable to patients with rheumatoid arthritis and systemic lupus erythematosus in our previous published cohorts (6, 8). Comparison of DA label prevalence among different patient cohorts is shown in Table 2.

**Table 1:** Demographics and clinical characteristics of HAE patients with or without DA labels.

	Total (*n* = 41)	With DA label (*n* = 9)	Without DA allergy (*n* = 32)	*P*-value
Male	20 (48.8%)	3 (33.3%)	17 (53.1%)	0.454
Age of HAE diagnosis (years)	37.9 ± 20.3	46.4 ± 11.6	35.5 ± 21.7	0.056
Symptomatic HAE	27 (65.9%)	9 (100%)	18 (56.3%)	**0.017**
Delay in diagnosis (years)	13.1 ± 14.7	23.8 ± 11.1	10.2 ± 14.3	**0**.**012**
Frequency of HAE attacks				
More than 1 attack per year	24 (58.5%)	9 (100%)	15 (46.9%)	**0**.**005**
More than 5 attacks per year	11 (26.8%)	7 (77.8%)	4 (12.5%)	**<0**.**001**

Drug allergy (DA). 2 cohorts; with DA label and without DA.

**Table 2:** Comparison of the prevalence of various drug allergy labels among different patient cohorts in Hong Kong.

	Hereditary angioedema (Current study) (*N* = 41)	Hospitalized patients in general medical wards (*N* = 3,641)	Ambulatory patients in general medical clinics (*N* = 3,540)	Rheumatoid arthritis (*N* = 1,286)	Systemic lupus erythematosus (*n* = 496)
Any drug allergy label	9 (24%)	510 (14%)	487 (14%)	287 (22%)	199 (40%)
Antibiotic allergy	7 (17%)	258 (7%)	247 (7%)	113 (9%)	129 (26%)
Beta-lactam allergy	6 (15%)	178 (5%)	170 (5%)	69 (5%)	82 (17%)
Non-steroidal anti-inflammatory drugs allergy	5 (12%)	—	78 (2%)	73 (6%)	30 (6%)

Among the 9 HAE patients with DA labels, 3 (33%) of them were male, with a median age of 49 (30–74) years. All 9 patients were symptomatic of HAE and their reported DA labels preceded their HAE diagnosis. There was a median delay of HAE diagnosis was 25 (7–37) years and the median duration of a DA label was 11 (0–18) years. Five of nine (56%) patients had more than 1 DA label and there was a total number of 16 DA labels. The majority (78%) of DA labels were reported as an immediate-type hypersensitivity reactions (HSR), and the remaining were either unknown or forgotten. Symptomatology of the index drug reactions were limited to swelling, angioedema, and hives/urticaria. No patients reported any history of suspected non-immediate-type HSR.

### Presence of DA label associated with delay in HAE diagnosis, likelihood of experiencing HAE attacks and rate of hospitalization

Patients with a DA label were more likely to be symptomatic with HAE (100% vs. 56.3%, *p* = 0.017). A DA label was also more likely associated with a delay in HAE diagnosis (23.8 ± 11.1 vs. 10.2 ± 14.3 years, *p* = 0.012). Patients carrying DA labels were more likely to have at least one (100% vs. 46.9%, *p* = 0.005), or frequent (≥5 per year) HAE attacks (77.8% vs. 12.5%, *p* < 0.05), as well as number of hospital admissions per year (3.78 ± 2.68 vs. 1.32 ± 2.61, *p* = 0.022).

### Use of alternative antibiotics and hospitalization days for patients with BL allergy labels

**Figure 1:**
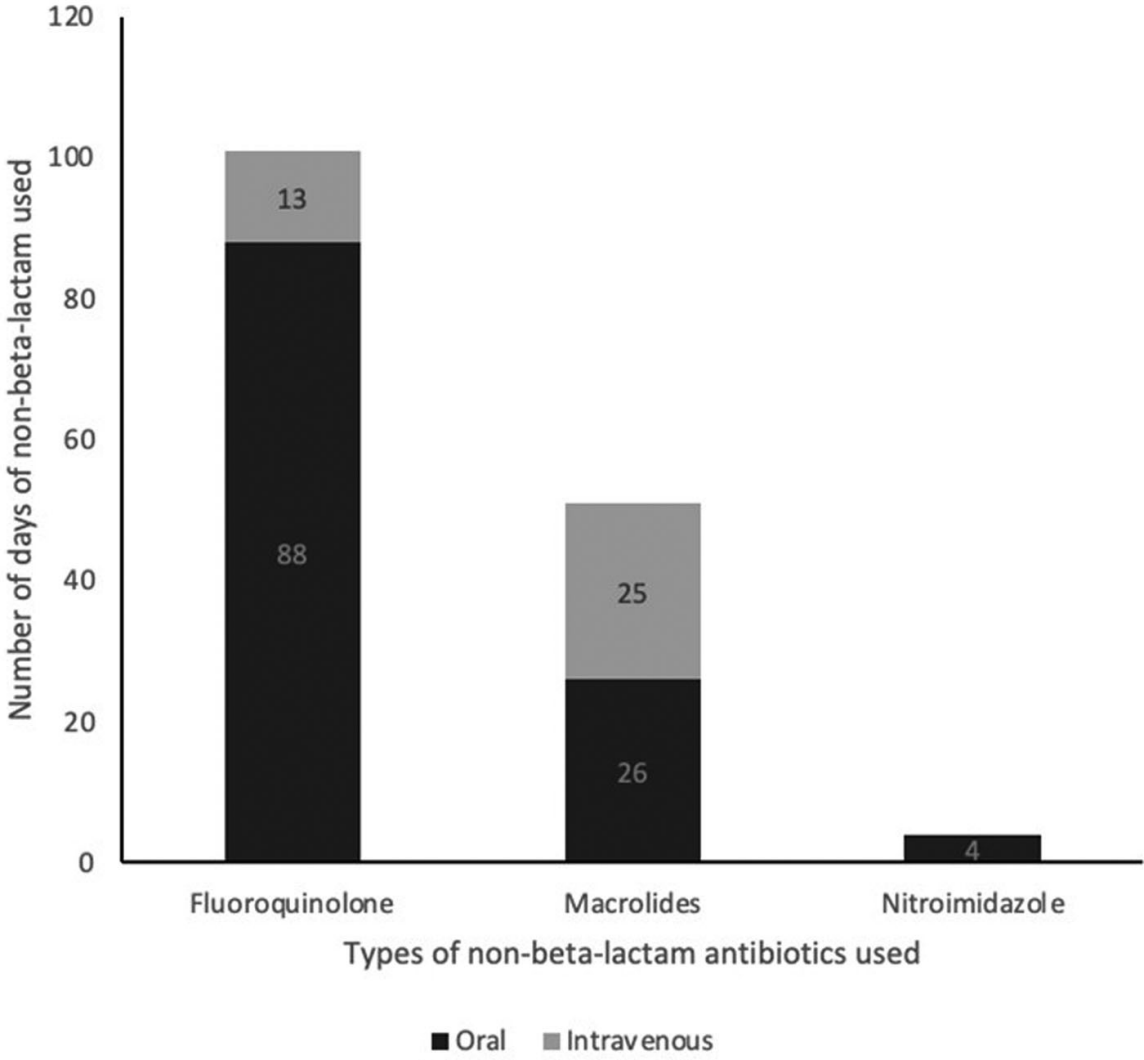
Total number of days of non-beta-lactam antibiotics used by HAE patients with beta-lactam allergy label.

The most common DA label was to BL (37.5%). Patients with BL allergy labels were also subject to receiving multiple courses of non-BL antibiotics. The most frequently used non-BL antibiotic alternative in patients with a BL allergy label were fluoroquinolones followed by macrolides (Figure 1). Days of hospitalization due to suspected infective causes within the duration being labelled ranged from 0 to 35 days, with a median of 8 days.

### High rate of misdiagnosed DA

**Figure 2:**
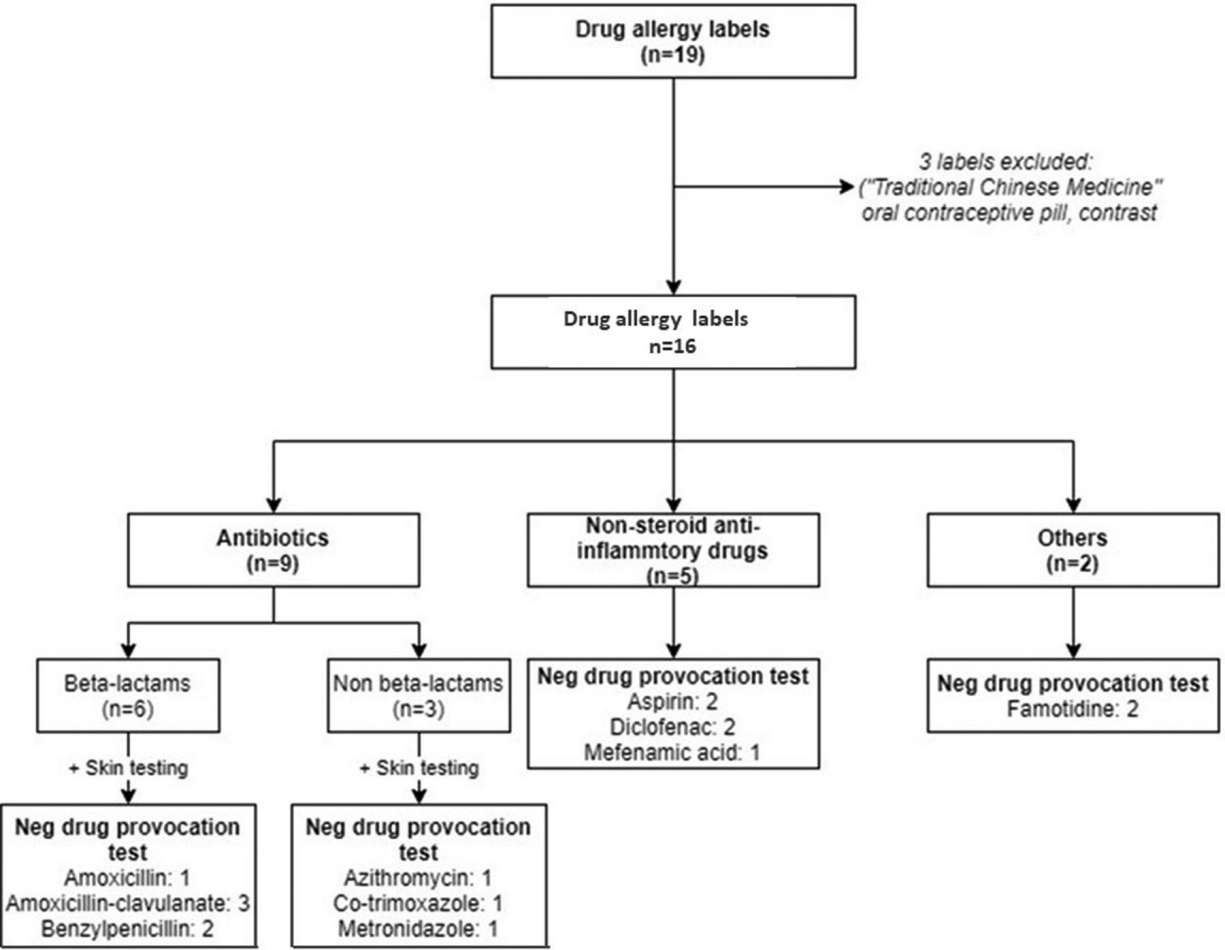
Allergy investigations and outcome of HAE patients with suspected drug allergy labels.

All 9 patients, with a total of 16 DA labels, underwent allergy testing negative results. All misdiagnosed DA labels were removed after confirmatory DPT were performed. Results of all DA investigations is shown in Figure 2 and summarized in Supplementary Table S1.

## Discussion

There has been a lack of emphasis on the importance of evaluating and revisiting suspected drug allergies in patients with HAE. There have been limited reports on DA labels of HAE patients prior to this study, and we present the first study investigating the impact and evaluation of DA labels among HAE patients. In clinical practice, DA labels in this specific cohort is highly relevant because of the difficulty many physicians have in differentiating the two entities. When comparing the prevalence of DA among other patient cohorts, DA labels among HAE patients are amongst the highest. HAE patients present with recurrent episodes of bradykinergic angioedema which is often mistaken for histaminergic angioedema, such as seen with immediate-type DA (3). This likely explains why there were no reports of non-immediate DA among our HAE cohort.

All patients with DA labels were symptomatic and likely erroneously labelled with DA during an HAE attack. Notably, we discovered that the presence of DA labels was associated with delay in HAE diagnosis. Especially with the lack of immunologists/allergists in Hong Kong, a diagnosis of suspected drug allergy is seldom challenged and therefore recurrent HAE attacks may have been misdiagnosed with DA instead. This would further compound the already-significant delay (average of 28 years) in HAE diagnosis. In a vicious cycle, a significant delay in diagnosis and continued undiagnosed HAE attacks would allow more opportunities for mislabelling of allergies to occur.

We also noticed a different pattern of DA labels among HAE patients, with an unexpectedly high number of reported of famotidine allergy (2/20 DA labels) given the rarity of reported cases before (15). We postulate that gastrointestinal HAE attacks could lead to increased use of H_2_ receptor antagonists and exposure to this drug class—which could lead to increased chance of sensitization (i.e., developing genuine DA) or opportunities for mislabelling. Such incorrect DA labels lead to unnecessary avoidance of indicated drugs in the future, further contributing to symptom severity and reduced quality of life. As in other allergy studies, it would be of great interest to study the impact of individual DA labels on HAE outcomes in the future. Understanding the unique pattern and prevalence DA labels among different disease cohorts is important to help prioritize scarce allergy resources toward drug de-labelling.

Erroneous labelling of BL allergy has been universally known and lead to multiple adverse consequences including increased length of stay and hospitalization, poorer outcomes in treatment of infections, increase risk of multi-drug resistant organisms and increased mortality (16, 17). We discovered that the detrimental effects of BL allergy labels were also seen among our HAE cohort. HAE patients with BL allergy labels were obliged to use of alternative antibiotics and associated with hospitalization. Since symptomatic HAE patients are at an even higher risk of hospitalization, this could increase drug exposure and subsequent sensitization and development of a genuine allergy. However, after workup we discovered that this is not the case and that most DA labels were incorrect. Furthermore, we discovered that DA labels were also significantly associated with frequency of HAE attacks, hospitalization and number of admissions per year among our cohort. This can be due to a multitude of reasons; for example, infections can both trigger and exacerbate HAE attacks, and therefore incorrect DA labels would restrict antibiotic choices and potential further exacerbating HAE severity. More severe HAE attacks would more likely warrant hospitalization as well as prolong length of stay. Incorrect antibiotic allergy labels may also limit the choice of oral antibiotics, and again increase the likelihood for hospitalization for administration of parental medications. The prolonged hospitalizations not only affect patients' comfort and quality of life, more healthcare resources would be required.

Given the additional adverse clinical consequences with HAE patients, we hope to emphasize the importance of accurate DA diagnosis among this susceptible cohort. We advocate that an effort for active and pre-emptive delabelling (i.e., actively working up suspicious DA labels even prior to recurrent need of implicated drugs) should be included in the optimal management of HAE. From our experience, a good opportunity would be to offer allergological testing following initial diagnosis of HAE especially as the patient is usually already seeing an immunologist/allergist during HAE workup. Failing that, physicians should take opportunity during clinic visits to discuss DA delabelling

There are several limitations to this study. HAE is a relatively rare disease and we had a small cohort of cases. It was therefore not possible to perform sub-analysis on the different classes of DA labels. All the index reactions reported in our cohort were mostly mild reactions only. Patients with non-immediate-type or severe HSR are under-represented in this cohort and the implications for these groups of allergies are uncertain. We would also recommend patients who fall under these groups to carry out DA delabelling as the discretion of the attending allergist or immunologist. Although rarely reported, there may have been a possible loss of sensitization for IgE-mediated drug allergies (with subsequent “resensitization” following DPT) leading to a “false negative” DPT. Although we have not encountered such phenomenon, despite many cases having received repeated courses of the culprit drugs thereafter, formal re-testing and future longitudinal studies would be useful. Also, the longitudinal impact and clinical outcomes for testing non-BL antibiotics remain to be investigated.

In conclusion, DA labels among HAE patients are prevalent but are frequently mislabelled and misdiagnosed. Misdiagnosed DA are associated with delay in HAE diagnosis as well as adverse clinical outcomes—such as increased frequency of HAE attacks and hospitalization rates. We advocate that immunologists/allergists should be consider pre-emptively review and workup every suspicious DA label, especially among our own HAE patients. Moreover, other non-drug related causes of angioedema e.g. HAE, should be excluded in patients with recurrent stereotypical attacks of angioedema or angioedema without concurrent urticaria.

## Data Availability

The original contributions presented in the study are included in the article/Supplementary Material, further inquiries can be directed to the corresponding author/s.
